# Cells and Fugu Response to Capsid of BFNNV Genotype

**DOI:** 10.3390/v15040988

**Published:** 2023-04-18

**Authors:** Mingguang Mao, Jielan Jiang, Jia Xu, Yumeng Liu, Haishan Wang, Yunxiang Mao

**Affiliations:** 1 Yazhou Bay Innovation Institute, Hainan Tropical Ocean University, Sanya 572022, China; maomingguang@foxmail.com (M.M.); jazz-123@163.com (H.W.); yxmao@hntou.edu.cn (Y.M.); 2 Key Laboratory of Utilization and Conservation for Tropical Marine Bioresources, Ministry of Education, Hainan Tropical Ocean University, Sanya 572022, China; 3 College of Fisheries and Life Sciences, Dalian Ocean University, Dalian 116023, China; doneharvar@163.com (J.X.); 15526842076@163.com (Y.L.)

**Keywords:** nervous necrosis virus, capsid, DNA vaccine, immune response

## Abstract

The nervous necrosis virus (NNV) of the BFNNV genotype is the causative agent of viral encephalopathy and retinopathy (VER) in cold water fishes. Similar to the RGNNV genotype, BFNNV is also considered a highly destructive virus. In the present study, the RNA2 of the BFNNV genotype was modified and expressed in the EPC cell line. The subcellular localization results showed that the capsid and N-terminal (1–414) were located in the nucleus, while the C-terminal (415–1014) of the capsid was located in the cytoplasm. Meanwhile, cell mortality obviously increased after expression of the capsid in EPC. EPC cells were transfected with pEGFP-CP and sampled at 12 h, 24 h and 48 h for transcriptome sequencing. There are 254, 2997 and 229 up-regulated genes and 387, 1611, and 649 down-regulated genes post-transfection, respectively. The ubiquitin-activating enzyme and ubiquitin-conjugating enzyme were up-regulated in the DEGs, indicating that cell death evoked by capsid transfection may be related to ubiquitination. The qPCR results showed that heat stock protein 70 (HSP70) is extremely up-regulated after expression of BFNNV capsid in EPC, and N-terminal is the key region to evoke the high expression. For further study, the immunoregulation of the capsid in fish pcDNA-3.1-CP was constructed and injected into the *Takifugu rubripes* muscle. pcDNA-3.1-CP can be detected in gills, muscle and head kidney, and lasted for more than 70 d post-injection. The transcripts of IgM and interferon inducible gene Mx were up-regulated after being immunized in different tissues, and immune factors, such as IFN-γ and C3, were also up-regulated in serum, while C4 was down-regulated one week after injection. It was suggested that pcDNA-3.1-CP can be a potential DNA vaccine in stimulating the immune system of *T. rubripes*; however, NNV challenge needs to be conducted in the following experiments.

## 1. Introduction

Nervous necrosis virus (NNV), the causative agent of viral encephalopathy and retinopathy (VER), is considered a highly destructive viral disease in aquaculture. It has infected more than 50 fish species, including a variety of farmed and wild marine fish and freshwater fish [1,2,3,4]. In particular, fish larvae are the more susceptible to NNV and the mortality rates are as high as 100% [1]. 

In recent years, asymptomatic infections of NNV have been reported in species other than fish, including the live baits brine shrimp (*Artemia salina*), rotifer (*Brachionus plicatilis*), shellfish, and loggerhead turtle (*Caretta caretta*) in Italy [5,6,7]. New research also found that invasive species are more susceptible than native species [8]. The expansion of host range greatly increases the harmfulness of the virus, while complex relationship between the virus and its susceptible host range and stability in the environment will inevitably affect species diversity in seawater environment [2,9].

NNV is a non-enveloped, about 30 nm icosahedral virus with two molecules, RNA1 and RNA2 of single-stranded positive-sense RNA, which are capped but not polyadenylated [10]. Usually, NNVs are classified into five genotypes based on the sequences of RNA2: red-spotted grouper NNV (RGNNV); barfin flounder NNV (BFNNV); tiger puffer NNV (TPNNV); striped jack NNV (SJNNV); and Turbot NNV (TNNV) [11,12]. Interestingly, RGNNV infects warm-water fish, such as groupers, Asian sea bass, and European sea bass, while BFNNV infects cold-water fish, such as Atlantic halibut, Atlantic cod, Pacific cod, and flounders [1,3,13,14,15]. Souto et al. [16] found that the C-terminal domain of the capsid is the key factor to determine the host specificity of an NNV. In addition, modification of the positions of 247 amino acids (Ser to Ala) and 270 amino acids (Ser to Asp) in the capsid of SJNNV and RGNNV strains reduced viral replication in sole neurons and sharply decreased the infection mortality of sea bass [17]. However, literature related to the capsid of the BFNNV genotype is still limited.

NNV control strategies remain a current concerned subject for the aquaculture industry. Chemical and physical treatments and NNV-free brood stocks are commonly taken to prevent NNV transmissions [18,19]. A traditional inactivated vaccine has been well studied, and the DNA vaccine is now popular in controlling fish disease [20,21,22]. Vaccines based on the capsid of the RGNNV type have been developed [23], but the BFNNV vaccine has still not been studied yet. In this study, we developed a plasmid DNA combined with the BFNNV capsid gene. The subcellular localization of BFNNV capsid was studied, and the cell survival and gene expression levels post-BFNNV capsid-transfected were determined. The N-terminal or C-terminal, which was responsible for the high expression of HSP70 in EPC cells was studied. The immunoregulation of *pcDNA3.1-CP* in vivo was further analyzed. We aimed to explore the pcDNA-3.1-CP to be a potential DNA vaccine.

## 2. Materials and Methods

### 2.1. Cell Lines and Fish 

EPC cells were provided by Prof. Qiya Zhang, Chinese Academy of Science, and cultured at 25 °C in medium 199 with 10% fetal bovine serum (FBS). Healthy *T. rubripes*, weighing approximately 200–300 g, were obtained from the Dalian Tianzheng Industry Co., Ltd., Dalian, China. The fish were maintained in a holding tank with a water temperature of 16–17 °C, and dissolved oxygen of about 6.84–7.80 mg/L was fed daily with fishing bait.

### 2.2. Plasmids Construction

**Figure 1:**
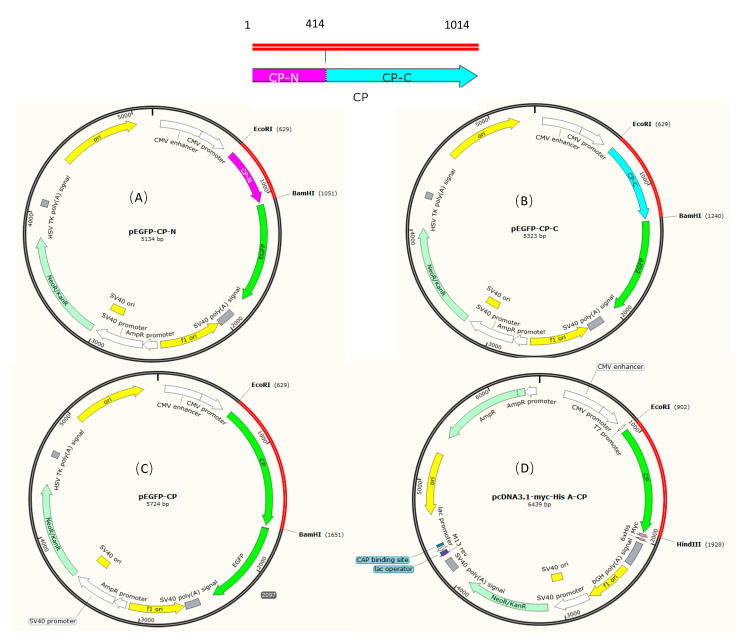
Plasmid construction of BFNNV capsid. (**A**) Map of pEGFP-CP-N. The N-terminal of capsid protein is colored pink; (**B**) Map of pEGFP-CP-C. The C-terminal of capsid protein is colored blue; (**C**) Map of pEGFP-CP with the tag of EGFP; (**D**) Map of pcDNA3.1-CP with tags of Myc and His.

Based on the sequence of the open reading frame (ORF) of BFNNV capsid (GenBank accession NO. KM576685), primers with restriction sites were designed (Table 1). Based on the abundance of antigen sites, RNA2 was divided into two fragments, namely, the N-terminal fragment (1–414 nt) and the C-terminal fragment (415–1014 nt), respectively. The N-terminal, C-terminal, and the whole RNA2 fragments were inserted into the empty plasmid pEGFP-N1, and named pEGFP-CP-N, pEGFP-CP-C and pEGFP-CP, respectively. The sequence of pEGFP-CP, pEGFP-CP-N and pEGFP-CP-C was confirmed by restriction enzyme digestions and DNA sequencing. The ORF of BFNNV capsid was cloned into the eukaryotic expression vector pcDNA3.1-myc-His A (Invitrogen, Waltham, MA, USA) using EcoR Ⅰ and Hind Ⅲ restriction enzyme sites to obtain the plasmid pcDNA3.1-CP, which will be used for a potential plasmid DNA vaccine in *T. rubripes.* The plasmids pEGFP-CP, pEGFP-CP-N, pEGFP-CP-C and pcDNA3.1-CP were purified using the Endo-free Plasmid Midi Kit (Tiangen Biotech, Beijing, China), and then stored at −20 °C until used (Figure 1).

### 2.3. Subcellular Localization of Modified Plasmids in EPC Cells

EPC cells were seeded into 6-well culture plates and grown in medium 199 supplemented with 5% fetal bovine serum (FBS). The cells were transfected with pEGFP-CP, pEGFP-CP-N and pEGFP-CP-C using Lipofectamine^®^ 3000 (Invitrogen) according to the manufacturer’s instructions, respectively. At 48 h post-transfection, the cells were rinsed with 1 × PBS (pH 7.4) and fixed with 4% paraformaldehyde for 30 min. Subsequently, the cells were permeabilized with 0.2% Triton X-100 for 15 min and stained with Hoechst 33258 (1 μg/mL) for 10 min. Finally, the cells were mounted with 50% glycerol and observed under a fluorescence microscope (Leica DM4B).

### 2.4. Cell Counts

Cells from the same cell bottle were distributed into two 24-well plates on average. In the first plate, four wells were set as the control group, four other wells were set as pEGFP-N1 transfection group, and another four wells were set as pEGFP-CP transfection group. The medium with dead cells was removed, and the living cells were counted 24 h post-transfection. The same method was conducted for the second plate 48 h post-transfection.

### 2.5. Gene Expression Responses of Cells to BFNNV CP

EPC cells were sampled at 12 h, 24 h and 48 h after cells were transfected with pEGFP-CP. At the same time, controls of the cells transfected with pEGFP-N1 were also sampled. Transcriptome sequencing and differential expressed genes (DEGs) analysis were conducted by Sangon Biotech (Shanghai, China). Genes were compared among nine public databases, including NCBI non-redundant protein sequences (NR), NCBI nucleotide sequences (NT), eukaryotic Ortholog Groups (KOG), Conserved Domain (CDD), Database Protein family (PFAM), a manually annotated and reviewed protein sequence database (SWISS-PROT), TrEMBL, Gene Ontology (GO), and Kyoto Encyclopedia of Genes and Genomes (KEGG). Gene expression between different groups was compared using the Fragment Per Kilobase Million mapped Reads method, and DEGs with a mean *p* ≤ 0.01 and expression ratio ≥ 2 were identified. Transcripts of a small subset of up- and down-regulated genes were evaluated by quantitative real-time PCR (qPCR) (Table 1), including Heat shock protein 70 (HSP70), ubiquitin-activating enzyme (UBA1), ubiquitin-conjugating enzyme (UBE2), Ras gene, MOB and DAP. An mRNA expression level of HSP70 was also detected in pEGFP-CP-N or pEGFP-CP-C transfected cells, respectively. 

### 2.6. Immunoregulation of pcDNA3.1-CP in T. rubripes

The plasmid pcDNA3.1-CP was diluted with sterile 1×PBS buffer to a concentration of 300 ng/μL. Twenty-four healthy *T. rubripes* were intramuscularly injected with 300 μL of pcDNA3.1-CP, while fish injected with pcDNA3.1 was used as the control group. One week later, *T. rubripes* were injected for the second time. After the second injection, the spleen, head kidney, muscle from the injection site and gills were collected 7 d post-injection (dpi) at 14 dpi, 42 dpi and 70 dpi. At the same time, blood was also collected at 7 dpi and 14 dpi, respectively. Blood was left to coagulate 1.5–2 h on ice and then centrifuged at 3000 g for 20 min at 4 °C. Sera was isolated and stored at −20 °C.

#### 2.6.1. The Retention Time of the Plasmid In Vivo

The genomic DNA of muscle, spleen, head kidney and gills were extracted using the TIANamp Marine Animals DNA Kit (Tiangen Biotech) with RNaseA (100 mg/mL) following the operating instructions. PCR was carried out with specific primers CP-F/R (Table 1). 

#### 2.6.2. Detection of Immune Factors after Plasmid Injection

Following the manufacturer’s instructions, the total RNAs of spleen, head kidney and gill samples were extracted using Trizol Reagent (Sangon Biotech), and 1 μg of RNA was reverse transcribed into the first strand cDNA using FastKing gDNA Dispelling RT SuperMix (Tiangen Biotech). A qPCR was used to evaluate the relative RNA amounts of immune response-related genes, including immunoglobulin M (IgM), myxovirus resistance 1 (Mx1) and tumor necrosis factor-alpha (TNF-α) genes. An SYBR Green real-time qPCR assay was performed using a StepOne real-time PCR system (Applied Biosystems, Waltham, MA, USA). The qPCR conditions were as follows: 50 °C for 2 min; 95 °C for 10 min; and 40 cycles of denaturing at 95 °C for 15 s, followed by annealing and primer extension at 60 °C for 1 min. All samples were tested in triplicates. Transcriptional levels of puffer fish immune genes were normalized by internal control gene β-actin and evaluated based on 2^−ΔΔCt^ method. The serum levels of C3, C4 and IFN-γ were detected using ELISA. According to the manufacturer’s instructions, the direct binding ELISA kit specially designed for fish (Shanghai Fankew Biotechnology Co., Ltd., Shanghai, China) was used for measurement. The results were described as trace amounts of C3, C4 or IFN-γ per 50 μL serum sample.

### 2.7. Statistical Analysis

The results were expressed as mean ± SEM. Student’s *t*-test was used for statistical comparison by SPSS 25.0. The level of significance was defined at *p* < 0.05.

## 3. Results

### 3.1. Capsid Localization and Cell Response

**Figure 2:**
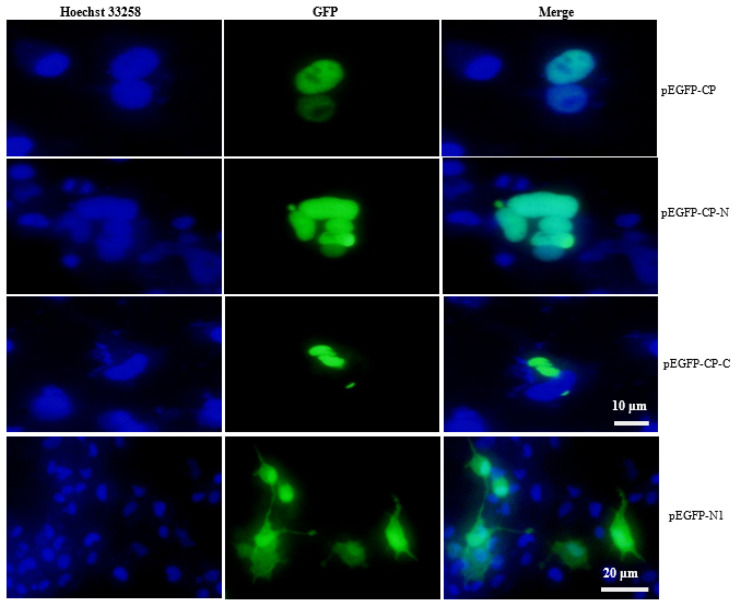
Subcellular localization of NNV-CP in EPC. Subcellular localization of CP in EPC cells transfected with pEGFP-CP-N, pEGFP-CP-C and pEGFP-CP. After allowing the cells to adhere for 48 h in 6-well plates, the nucleus was stained with Hoechst 33258, and fluorescent signals were observed under a fluorescence microscope.

**Figure 3:**
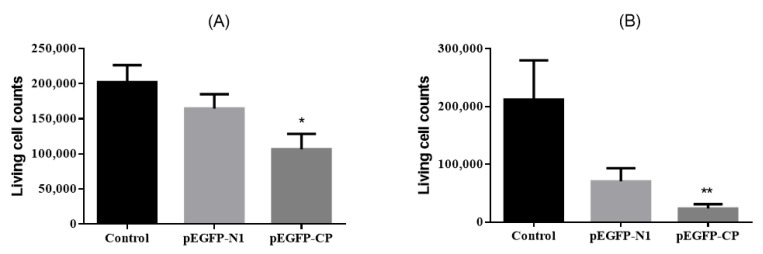
Living cell counts after transfection. (**A**) 24 h post transfection, (**B**) 48 h post transfection (*: *p* < 0.05; **: *p* < 0.01).

As shown in Figure 2, NNV-CP was mainly located in the nucleus, and the N-terminal was located in the nucleus while the C-terminal was mainly located in the cytoplasm. Interestingly, the cells seem to be dying when CP was expressed, as the cell nucleus becomes round and condensed, and the cells no longer adhere to the culture dish (see in Supplementary File Figure S1). In addition, the number of living cells gradually decreased (Figure 3).

### 3.2. Gene Expression Responses of Cells to BFNNV Capsid

**Figure 4:**
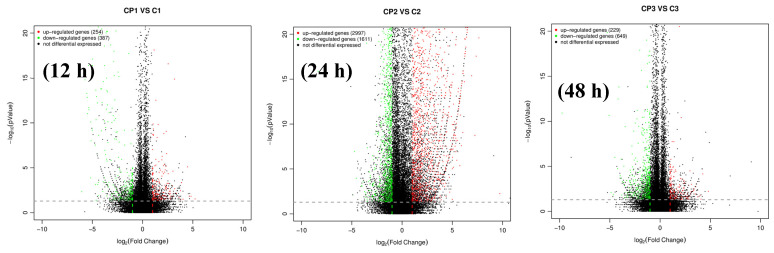
DE volcano and DE gene barplot after CP overexpression. CP1, CP2, and CP3 were at 12 h, 24 h and 48 h post-infection with pEGFP-CP, respectively; C1, C2, and C3 were tested at 12 h, 24 h and 48 h post-infection with pEGFP-N1 as control, respectively.

**Figure 5:**
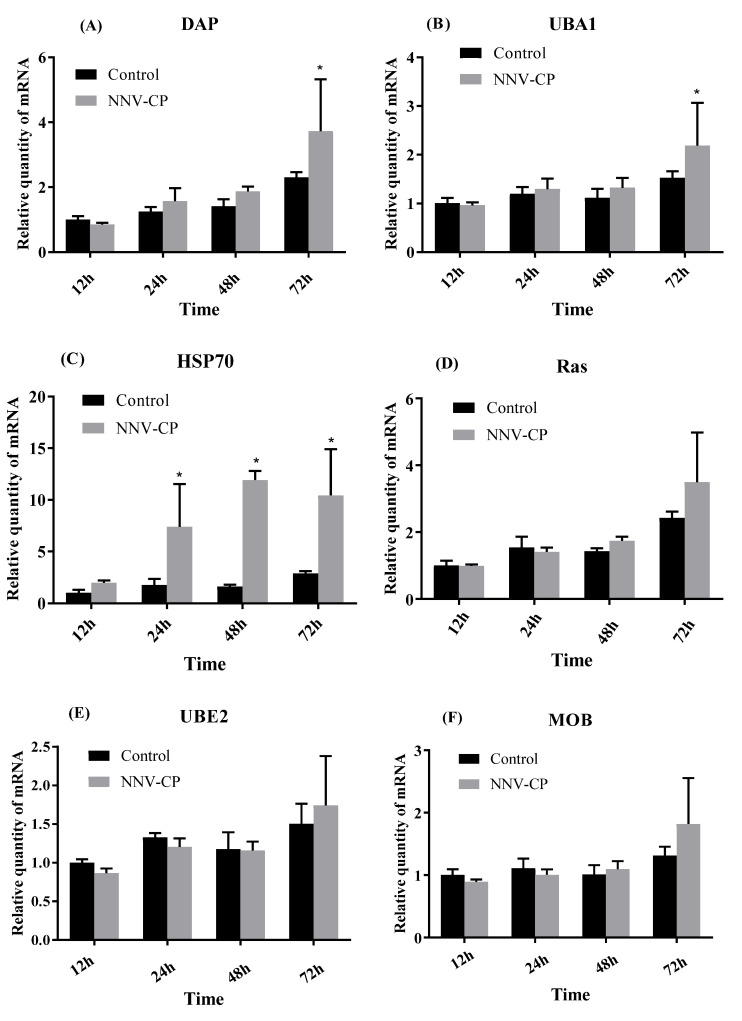
Transcriptional levels of DAP (**A**), UBA1 (**B**), HSP70 (**C**), Ras (**D**), UBE2 (**E**) and MOB (**F**) after pEGFP-CP transfection (*: *p* < 0.05).

**Figure 6:**
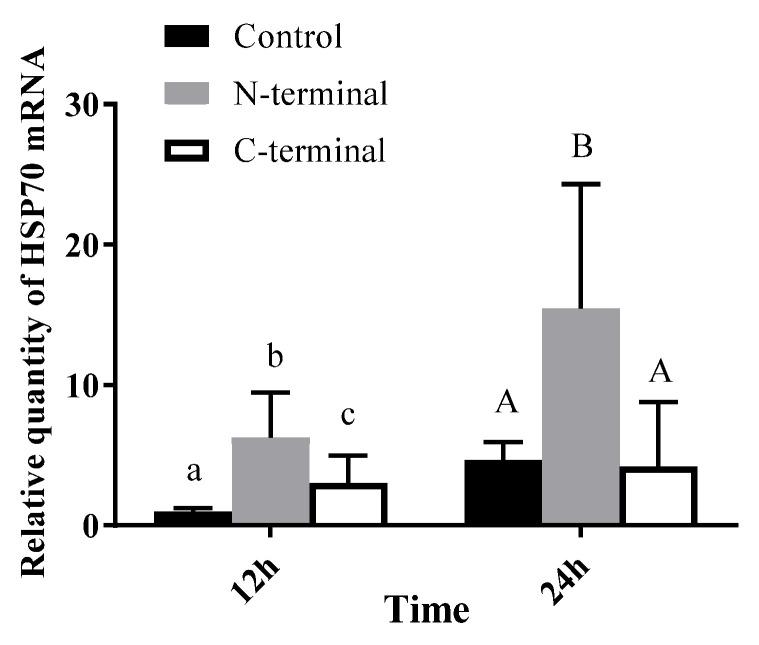
mRNA expression level of HSP70 after pEGFP-CP-N (N-terminal) or pEGFP-CP-C (C-terminal) transfection. Different letters represent significant differences between groups (*p* < 0.05).

EPC cells were transfected with pEGFP-CP, and cells were sampled after 12 h, 24 h and 48 h, respectively. Compared with the controls of each timepoint, there are 254 up-regulated genes and 387 down-regulated genes at 12 h post-transfection, 2997 up-regulated genes and 1611 down-regulated genes at 24 h post-transfection, and 229 up-regulated genes and 649 down-regulated genes at 48 h post-transfection (Figure 4). The most significant DEGs were listed in Table 2. Heat shock protein 70 (HSP70), ubiquitin-activating enzyme (UBA1), ubiquitin-conjugating enzyme (UBE2), Ras gene, MOB and DAP were tested using qPCR. HSP70 was significantly up-regulated at 24 h to 72 h post-transfection of pEGFP-CP. DAP and UBA1 were up-regulated at 72 h post-transfection (Figure 5). Interestingly, the N terminal of BFNNV CP is the key domain to induce high expression of HSP70 as shown in Figure 6.

### 3.3. Plasmid Tested in Various Tissues after Injection

*T. rubripes* were injected with pcDNA3.1-CP twice at a one-week interval. Muscle, spleen, head kidney and gill tested positive for NNV-CP signal using PCR at 7 dpi, 14 dpi, 42 dpi and 70 dpi, respectively (Supplemented File Figure S1). The target PCR products were sequenced to confirm the NNV-CP segment.

### 3.4. Immune Response of T. rubripes after Plasmid Injection

**Figure 7:**
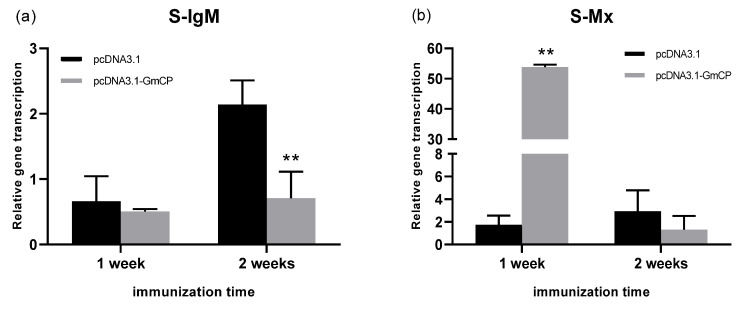

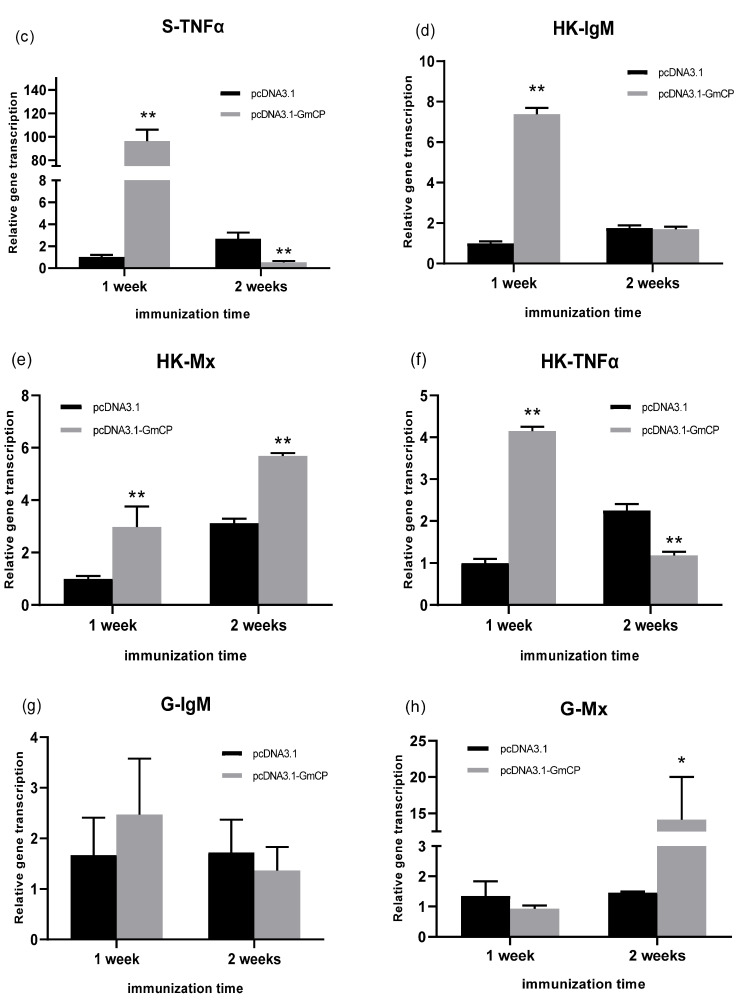

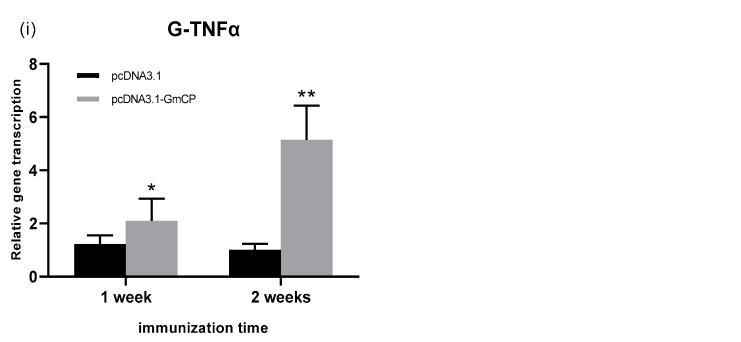
Expression of immune-related genes in different tissues after two injections of pcDNA3.1-CP (*: *p* < 0.05; **: *p* < 0.01). IgM, Mx and TNF-α in the spleen (S), head kidney (HK) and gill (G) were tested separately using qPCR (from (**a**) to (**i**)).

IgM was significantly up-regulated in the head kidney one week after the second injection (Figure 7d). The transcript levels of interferon-inducible gene Mx increased obviously in the spleen, head kidney and gill after the second injection (Figure 7b,e,h). The transcript levels of TNF-α were up-regulated in the spleen and head kidney one week after the second injection but decreased at two-week post-injection (Figure 7c,f). TNF-α were up-regulated obviously in the gill after immunization (Figure 7i).

**Figure 8:**
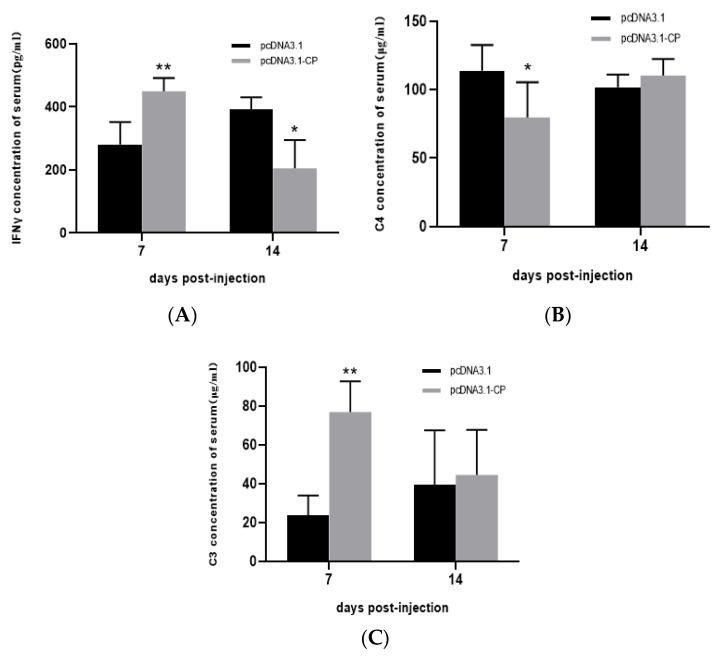
Cytokine levels in serum using ELISA after two injections of pcDNA3.1-CP (*: *p* < 0.05; **: *p* < 0.01).

After the second immunization, the serum levels of IFN-γ, complement factor C4 and C3 were detected using ELISA. The results showed that IFN-γ increased after one week, but decreased at two weeks post-second injection (Figure 8A). C3 was up-regulated (Figure 8B), while C4 was down-regulated at one-week post-injection (Figure 8C).

## 4. Discussion 

The genome structure of NNV is simple, consisting of two positive RNAs [16]. RNA1 encodes RNA polymerase. RNA2 codes for the capsid protein (CP), and each of the CP (42 kDa) consists of the following domains: an arginine-rich N-terminal domain (residues 34–51) that is responsible for recruiting the RNA during encapsulation; a shell domain (residues 52–213; S-domain) that forms the cage for the encapsulated RNA and contains calcium-binding structures which seem to be essential for virus assembly; a flexible linker region (residues 214–220); and a protrusion domain (residues 221–338; P-domain), including the hypervariable region of the protein, is involved in the interaction with the host cell surface, and is also responsible for the trimerization of the protein [24]. Therefore, the capsid of NNV is considered one of the efficient antigens.

The RGNNV capsid has been studied extensively. A total of 43 amino acid residues in the N-terminal of RGNNV capsid were required to import EGFP-protein into the nucleolus. A total of 20 amino acids from position 14 to 33 of the N-terminal were the domain of nucleolus localization [25]. Iwamoto et al. [26] have revealed that the host specificity of NNV depends on RNA2 by exchanging the RNA2 gene between SJNNV and RGNNV. RNA1 of BFNNV isolated from *Atlantic halibut* was examined for its role in cells [27], but the mechanism of BFNNV RNA2 action on cells was still unclear. In the present study, CP of BFNNV was proved to localize in the nucleus, and nuclear localization is determined by the N-terminal of RNA2. The result is similar to that of RGNNV capsid. 

Overexpression of BFNNV capsid would lead to high mortality of the EPCs (data were not shown in the results). Using next-generation sequencing, some interesting DEGs were discovered after capsid overexpression. After qPCR checking, HSP70 was up-regulated significantly after BFNNV capsid expression from 24 h to 72 h. It was reported that grouper heat shock cognate protein 70 (GHSC70) participates in the NNV entry of GF-1 cells, likely functioning as an RGNNV receptor or coreceptor protein, while marine medaka HSP90ab1 was combined with RGNNV as a potential receptor [28,29]. Recent evidence of *Lateolabrax japonicus* heat shock protein HSP90ab1 provides a novel insight into the relationship between RGNNV receptors and autophagy [30]. In addition, HSP70 is the key factor together with CHIP inducing the ubiquitin–proteasome system (UPS) and the autophagosome–lysosome pathway [31]. Meanwhile, UBA1 was also detected with high expression at 72 h of BFNNV CP overexpression, and cells were dying at this time point, with high expression of apoptosis-related gene DAP [32]. In the present study, we also proved that the C-terminal of RNA2 is the key region to evoke a high expression of HSP70. The relationship between HSP70 and BFNNV needs to be further examined which may help clarify the pathogenesis of NNV.

DNA vaccine is an effective protection strategy against virus disease [22]. Immunization by an antigen-encoding DNA in the fishery was approved for commercial sale in Canada against a Novirhabdovirus infection in fish, but it was limited in other countries [33]. The pcDNA3.1 as an expression vector is used widely in DNA vaccine preparation [34,35]. In the present study, an open reading frame of BFNNV RNA2 was inserted into pcDNA3.1 successfully. Seven days post-injection, *pcDNA3.1-CP* was detected in muscle, head kidney, spleen and gill using PCR, and 70 days later, the PCR signal was still positive even though it was becoming weaker. It was suggested that *pcDNA3.1-CP* can be transmitted around the fish body and it can last for a long time, which is similar to the reported duration [36]. After immunization, the interferon-induced Mx gene was induced significantly, indicating that fish was in a virus-defending status, but IgM was not up-regulated obviously. Evidence showed that NNV-neutralizing antibodies (IgM) might not be necessary for the protection of convalescent fish against NNV re-infection after a previous NNV infection [37]. After immunization, immune factors and complement systems were also influenced, but the mechanism should be further examined as the vaccine is exogenetic for immunized fish. BFNNV-CP is lethal to EPC cells, so it may be harmful to fish tissue to some degree, but the immune system can be stimulated effectively. Therefore, NNV challenge needs to be conducted in the following experiments.

## Figures and Tables

**Table 1:** Primers used in the experiment.

Name	Seq. 5′→3′	Purpose
P1592F	GGAATTCATGGTACGCAAAGGGAATAAG	Including EcoRI and BamHI restriction sites for pEGFP-CP construction
P1592R	CGGGATCCCGGTTTCCCGAGTCAGCCCGGGTG	
P1924F	CGGAATTCATGGTACGCAAAGGGAATAAGAAAT	Including EcoRI and BamHI restriction sites for pEGFP-CP-N construction
P1924R	CGGGATCCCGGAAGGTGTGGTCGCTGTCAGTTGG	
P1925F	CGGAATTCATGGACGCAATTCAAGCGACTCGTG	Including EcoRI and BamHI restriction sites for pEGFP-CP-C
P1925R	CGGGATCCCGGTTTCCCGAGTCAGCCCGGGTGAAG	
PEGFP-N1	CGTCGCCGTCCAGCT CGACCAG	universal primer for pEGFP-N1
HSP70-F1	ATTGGAGATGCTGCTAAA	qPCR for Hsp70 in EPC cells
HSP70-R1	GACTGCACTACTGGGTCA	
Ras-F1	TTCTGGGTGAAGGAGTTA	qPCR for Ras in EPC cells
Ras-R1	TCCTGAACATCGTGGTAG	
UBA1-F	CCCGAAAGTCCGCATTAC	qPCR for UBA1 in EPC cells
UBA1-R	AGCATTTGCCACTCCATC	
DAP-F1	GTCGTCACTAAGGGAGATAAA	qPCR for DAP in EPC cells
DAP-R1	TGGTGGATGTGCTGGTTG	
UBE2-F1	AATGGCAGCATCTGTTTG	qPCR for UBE2 in EPC cells
UBE2-R1	AATGGATCATCTGGGTTT	
MOB-F1	CACTGGGCTGATGGAACT	qPCR for MOB in EPC cells
MOB-R1	TGAGCGTAAACACGGAAG	
Eactin-F	GGCACTGCTGCTTCCTC	Internal control gene of qPCR in EPC cells
Eactin-R	ACCGCAAGACTCCATACCC	
β-actin-F	ATCCGTAAGGACCTGTATGC	Internal control gene of qPCR in *T. rubripes*
β-actin-R	AGTATTTACGCTCAGGTGGG	
qIgM-F	GTCATCATCAATCCCAAGC	qPCR for IgM in *T. rubripes*
qIgM-R	CCTCGTCCTCCCACCAAAT	
qMx-F	AGCCTGGTGGTTGTTCCG	qPCR for Mx in *T. rubripe*
qMx-R	AGATACCCAAAGTCCGTTC	
qTNFα-F	CTTTCCGAGTGACTTGCG	qPCR for TNF-α in *T. rubripe*
qTNFα-R	AGTGGTCTCAGTGCCGATG	
CP-F	TTCAAGCGACTCGTGGTG	qPCR for RNA2 gene detection
CP-R	CGCAGGTGTTCCCGTATT	
T7-F	CCAGCGTAGTTCGGTCCTC	Universal primer T7
T7-R	GCTTCCTTTCGGGCTTTGT	

Note: The underlines represent the restriction sites of the restriction enzymes.

**Table 2:** NR/NT annotation of the most significant DEGs.

Transcript id	*p* Value	q Value	Up-/Down-Regulated	NR/NT
TRINITY_DN25567_c4_g1	0	0	up	gi|808157850|gb|AKC98253.1|capsid protein
TRINITY_DN26629_c1_g3	0	0	up	gi|152956217|emb|CT956064.14|Zebrafish DNA sequence from clone CH211-248K16 in linkage group 7
TRINITY_DN22595_c2_g1	0	0	up	gi|108767286|ref|YP_636112.1|cytochrome c oxidase subunit II
TRINITY_DN26629_c2_g1	0	0	up	gi|954431710|gb|KRY81076.1|Uncharacterized protein T4D_4496
TRINITY_DN26045_c0_g3	0	0	up	gi|685042682|emb|LN591207.1|*Cyprinus carpio* genome assembly common carp genome, scaffold 000028969
TRINITY_DN26590_c3_g2	0	0	down	gi|688611381|ref|XP_009295045.1|filamin-A isoform X1
TRINITY_DN26695_c4_g1	0	0	up	gi|148357120|ref|NP_001091866.1|uncharacterized protein LOC100037361
TRINITY_DN26734_c0_g1	0	0	up	gi|1024953445|ref|XM_016442040.1|low-density lipoprotein receptor-related protein 1-like
TRINITY_DN25436_c3_g3	0	0	up	gi|914726992|gb|AKV94009.1|heat shock 70 kDa protein
TRINITY_DN26629_c1_g2	4.10 × 10^−307^	2.45 × 10^−303^	up	gi|954411357|gb|KRY64374.1|hypothetical protein T4A_8672
TRINITY_DN23848_c0_g1	5.70 × 10^−119^	7.18 × 10^−116^	down	gi|1025064867|ref|XP_016335574.1|palladin-like
TRINITY_DN24829_c0_g10	1.95 × 10^−102^	4.42 × 10^−99^	up	gi|632986665|ref|XP_007910363.1|serine/arginine-rich splicing factor 10-like
TRINITY_DN18738_c0_g1	1.90 × 10^−96^	1.80 × 10^−93^	up	gi|34538600|ref|NP_904330.1|cytochrome c oxidase subunit I (mitochondrion)
TRINITY_DN24829_c0_g10	1.94 × 10^−93^	1.75 × 10^−90^	up	gi|632986665|ref|XP_007910363.1|serine/arginine-rich splicing factor 10-like
TRINITY_DN23725_c2_g2	6.54 × 10^−91^	5.83 × 10^−88^	down	gi|1025042229|ref|XP_016327971.1|integral membrane protein 2B-like
TRINITY_DN26608_c1_g2	1.73 × 10^−87^	1.48 × 10^−84^	up	gi|1020496620|ref|XP_016086939.1|dnaJ homolog subfamily A member 4-like
TRINITY_DN20834_c6_g2	6.94 × 10^−85^	1.20 × 10^−81^	down	gi|576080555|ref|NP_001276655.1|glyceraldehyde-3-phosphate dehydrogenase isoform 1
TRINITY_DN24829_c0_g17	2.93 × 10^−79^	4.38 × 10^−76^	down	gi|632986665|ref|XP_007910363.1|serine/arginine-rich splicing factor 10-like
TRINITY_DN23468_c2_g7	2.10 × 10^−77^	3.00 × 10^−74^	down	gi|803119291|ref|XP_012040812.1|elongation factor 1-alpha 1-like isoform X1
TRINITY_DN26618_c3_g2	1.15 × 10^−67^	7.49 × 10^−65^	down	gi|496216328|ref|WP_008930570.1|RNA-directed DNA polymerase
TRINITY_DN25524_c1_g1	4.82 × 10^−52^	2.11 × 10^−49^	up	gi|1025155852|ref|XP_016417438.1|zinc finger protein 501-like
TRINITY_DN19302_c0_g3	6.96 × 10^−50^	5.38 × 10^−47^	down	gi|38649320|gb|AAH63174.1|Eno1 protein
TRINITY_DN20700_c0_g1	6.10 × 10^−47^	4.27 × 10^−44^	down	gi|564364021|ref|XP_006243250.1|pyruvate kinase PKM isoform X2
TRINITY_DN26231_c3_g15	4.76 × 10^−43^	1.50 × 10^−40^	down	gi|1025371162|ref|XP_016397846.1|E3 ubiquitin–protein ligase RNF185-like
TRINITY_DN24783_c2_g1	1.33 × 10^−26^	3.48 × 10^−25^	down	gi|1020485608|ref|XP_016148398.1|ubiquitin-conjugating enzyme E2 (UBE2) R2
TRINITY_DN23560_c2_g9	7.58 × 10^−19^	1.83 × 10^−16^	up	gi|1025199235|ref|XM_016570095.1|MOB kinase activator 3C-like
TRINITY_DN22460_c4_g2	2.65 × 10^−17^	5.71 × 10^−15^	up	gi|528517584|ref|XP_005162108.1|PREDICTED: ubiquitin-like modifier-activating enzyme 1 (UBA1)
TRINITY_DN22980_c2_g2	4.60 × 10^−16^	7.27 × 10^−15^	up	gi|1025171303|ref|XM_016502395.1|ubiquitin-conjugating enzyme E2 (UBE2) E1-like
TRINITY_DN21121_c2_g4	6.63 × 10^−16^	5.87 × 10^−14^	up	gi|1020505514|ref|XP_016091700.1|Ras-related protein Rap-1b
TRINITY_DN24478_c1_g2	3.93 × 10^−9^	3.81 × 10^−8^	down	gi|1025190993|ref|XP_016423750.1|death-associated protein (DAP) kinase 2-like

## Data Availability

The data presented in this study are available on request from the corresponding author.

## Supplementary Materials

The following supporting information can be downloaded at: https://www.mdpi.com/article/10.3390/v15040988/s1, Figure S1: EPC cells transfected with pEGFP-N1 (A) and pEGFP-CP (B).
