# Supplementary material for: Cells and Fugu Response to Capsid of BFNNV Genotype
Source: Viruses. 2023 Apr 18;15(4):988. doi: 10.3390/v15040988 (PMC10142826; doi:10.3390/v15040988)
Supplement: Supplementary file 1 [file viruses-15-00988-s001.zip › viruses-2338996-supplementary.pdf]

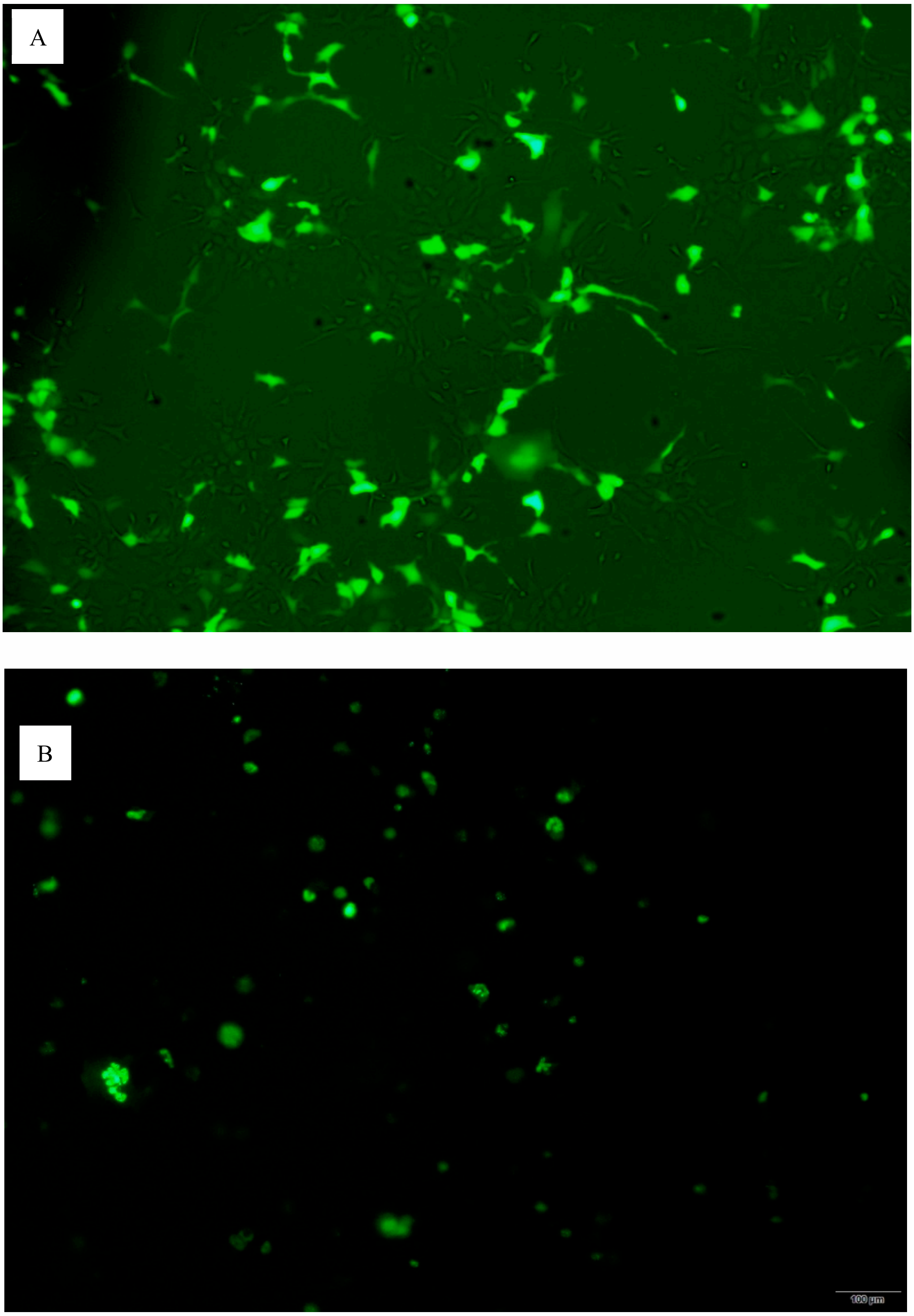

Supplementary Figure S1 EPC cells transfected with pEGFP-N1 (A) and pEGFP-CP (B)

Obviously, the normal cell seems dendritic as shown in the control group. With the capsid expressing in the EPC, the cell nucleus seems getting round and condensation for pieces and dying gradually as the cell never adheres to the culture dish. Dying cells are not on the same focal plane, so some appear blurry.
